# Exploring Micelles and Nanospheres as Delivery Systems for Phenothiazine Derivatives in Cancer Therapy

**DOI:** 10.3390/pharmaceutics16121597

**Published:** 2024-12-16

**Authors:** Katarzyna Jelonek, Monika Musiał-Kulik, Małgorzata Pastusiak, Aleksander Foryś, Andrzej Zięba, Janusz Kasperczyk

**Affiliations:** 1 Centre of Polymer and Carbon Materials, Polish Academy of Sciences, 34 Curie-Skłodowskiej St., 41-819 Zabrze, Poland; mmusial@cmpw-pan.pl (M.M.-K.); mpastusiak@cmpw-pan.pl (M.P.); aforys@cmpw-pan.pl (A.F.); 2 Department of Organic Chemistry, Faculty of Pharmaceutical Sciences in Sosnowiec, Medical University of Silesia, 4 Jagiellońska St., 41-200 Sosnowiec, Poland; zieba@sum.edu.pl; 3 Department of Biopharmacy, Faculty of Pharmaceutical Sciences in Sosnowiec, Medical University of Silesia, 8 Jedności St., 41-200 Sosnowiec, Poland

**Keywords:** phenothiazine, phenothiazine derivatives, drug delivery system, nanoparticles, nanocarriers, anticancer drugs

## Abstract

**Objectives**: Cancer remains one of the leading causes of death worldwide, and thus, there is a need for the development of innovative and more effective treatment strategies. The aim of the study was to evaluate two types of nanoparticles—nanospheres and micelles—obtained from PLA-based polymers to discover their potential for delivering four types of phenothiazine derivatives. **Methods**: The morphology, drug-loading properties, cytocompatibility, hemolytic properties and anticancer activity were analyzed. **Results**: The micelles exhibited significantly higher drug-loading properties, release process and cytotoxic activity against cancer cells compared to the nanospheres. The micelles containing 5-methyl-12*H*-quino[3,4-*b*][1,4]benzothiazinium chloride with an OH group as a substituent in the 10-position of the quinobenzothiazine ring showed the highest drug-loading content, the most efficient drug release, the lowest hemolytic activity and the most significant cytotoxic effect against HeLa cells. **Conclusions**: The conducted study enabled the development of a delivery system for the new anticancer compound and showed that the choice of drug carrier has a crucial effect on its cytotoxic potential against cancer cells.

## 1. Introduction

**Scheme 1:**
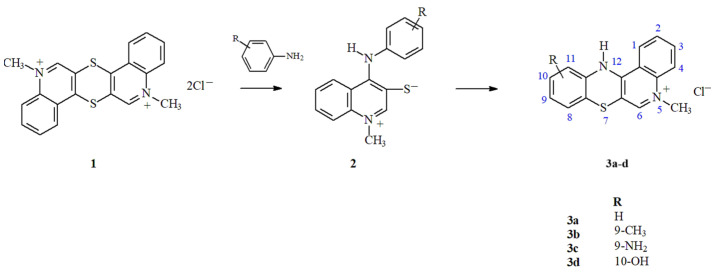
Synthesis of 5-methyl-12*H*-quino[3,4-*b*][1,4]benzothiazinium chlorides **3a**–**d**.

Phenothiazines are an interesting class of heterocyclic compounds possessing a tricyclic dibenzo-[1,4]-thiazine ring system with high therapeutic potential [1]. Phenothiazine derivatives may be even more effective in the treatment of anticancer diseases. An original method has been developed for the synthesis of azaphenothiazine derivatives that enables the formation of compounds containing specific substituents at different positions of the tetracyclic quinobenzothiazinium system [2]. Briefly, the method involves the reaction of thioquinanthrenediinium bis-chloride (**1**) with substituted isomeric anilines, which leads to the formation of a betaine system with the structure of 1-methyl-4-(phenylamino)quinoline-3-thiolate (**2**) as the intermediate product. As a result of its cyclization, a thiazine ring is formed (Scheme 1). Controlling the parameters of the cyclization reaction enables the introduction of various types of substituents in the 9, 10 and 11 positions of the quino[3,4-*b*][1,4]benzothiazine scaffold. Using this synthetic method, derivatives **3a**–**d**, containing different types of substituents (CH_3_, NH_2_, OH) in the quinobenzothiazinium system, were obtained.

The analysis of the cytotoxic activity of the 5-methyl-12*H*-quino[3,4-*b*][1,4]benzothiazinium chlorides (Scheme 1) against human colon carcinoma (Hct11) and Lewis lung carcinoma (LLC) cell lines showed a structure–activity relationship [2]. The greatest activity was exhibited by the compound with substituents in the 9- and 10-positions of the quinobenzothiazine ring and by the compound without substituents. However, along with the development of novel compounds with anticancer potential, it is also necessary to develop an appropriate drug delivery system (DDS). Systemic anticancer chemotherapy has some disadvantages, because small doses of the drug reach effective intratumoral concentrations, but higher doses, which cause significant tumor inhibition, may also increase toxicity to healthy cells [3]. Therefore, various strategies and DDSs are developed to avoid toxicity, control drug release and protect it from early elimination, extending circulation time, which increases tumor exposure to anticancer drugs.

One of the most extensively studied types of carriers of anticancer drugs are nanoparticles (NP). Compared to conventional drugs, nanoparticle-based DDSs have shown many advantages in cancer treatment, such as improved pharmacokinetics, stability, biocompatibility, enhanced permeability and retention effect, reduction of side effects and drug resistance [4]. However, the development of the optimal DDS is quite complex, and many factors that have an impact on their functionality need to be considered [5,6]. Liposomes based on dipalmitoylphosphatidylcholine (DPPC) have already been developed for co-encapsulation of doxorubicin (DOX) and 9-(*N*-piperazinyl)-5-methyl-12(*H*)-quino[3,4-*b*][1,4]benzothiazinium chloride [7]. Polymeric nanoparticles are more stable than liposomes, and their preparation methods are easy to reproduce [8]. Therefore, we have selected two types of NP—nanospheres (NS) and micelles (M)—obtained from PLA-based polymers to discover their potential for delivering four types of phenothiazine derivatives (**3a**–**d**, Scheme 1).

The aim of the study was to evaluate two types of nanoparticles—nanospheres and micelles—obtained from PLA-based polymers to discover their potential for delivering four types of 5-methyl-12*H*-quino[3,4-*b*][1,4]benzothiazinium chlorides with anticancer potential (Scheme 1). Our previous study showed that the functionality of the PLA-based injectable delivery systems strongly depends on the type of DDS and its polymeric composition [9].

## 2. Materials and Methods

### 2.1. Preparation of Nanoparticles

#### 2.1.1. Preparation of Nanospheres

Drug-free and drug-loaded NS were prepared using the emulsification technique. Poly(d,l-lactide-*co*-glycolide) was synthesized in bulk, with the use of Zr(Acac)_4_ as a non-toxic initiator, which was used to form the NS. Briefly, 125 mg of polymer was dissolved in 4 mL of methylene chloride. Then, 15 mg of drug (5-methyl-12*H*-quino[3,4-*b*][1,4]benzothiazinium chlorides: **3a**, **3b**, **3c** or **3d**) was dissolved in a mixture of methylene chloride and methanol (2:1 *v*/*v*). The drug solution and polymer solution were mixed and added dropwise to 50 mL of an ice-cold 5% polyvinyl alcohol (PVA) solution (*w*/*v*) and emulsified at 20,500 rpm for 2 min. The emulsion was gently stirred at room temperature (RT) overnight to evaporate the methylene chloride. The NS were collected by centrifugation at 10,000 rpm for 10 min. Then, they were washed three times with deionized water following centrifugation at 5000 rpm. The final NS were lyophilized and stored at 4 °C until further analysis.

The obtained nanospheres were marked as NS—drug-free nanospheres—and NS/3a, NS/3b, NS/3c or NS/3d-nanospheres loaded with **3a**, **3b**, **3c** or **3d**, respectively.

#### 2.1.2. Preparation of Micelles

The micelles (M) were obtained from poly(l-lactide)-polyethylene glycol (PLLA-PEG) (RuixiBiotech Co., Ltd., Xi’an, China) using the co-solvent evaporation method [10]. The number-average molar mass (Mn) of PLLA was 3000 Da, and the Mn of the PEG block was 5000 Da. The polymer was dissolved in chloroform (5 *w*/*v*%) and mixed with 5 mL of deionized water under vigorous stirring. The micellar solutions were left at RT for 24 h for solvent evaporation. The blank micelles were filtered through syringe filters (0.8 μm) to remove the precipitated polymer, then frozen at −80 °C and lyophilized.

To encapsulate a drug (**3a**, **3b**, **3c** or **3d**) in micelles, each compound was dissolved in ethanol (2 *w*/*v*%), and 50 μL of drug solution was added to a micellar solution and stirred magnetically. The initial (theoretical) drug content was 20 *w*/*w*%. The vials were left at RT for 24 h for solvent evaporation in a laminar box. Subsequently, the solution was centrifuged at 3000 rpm for 5 min to separate the unloaded drugs. The supernatant was collected, frozen, lyophilized and stored at 4 °C for further analysis.

The obtained micelles were marked as M—drug-free micelles—and M/3a, M/3b, M/3c or M/3d—micelles loaded with **3a**, **3b**, **3c** or **3d**, respectively.

### 2.2. Microscopic Analysis

The freeze-dried NS were subjected to morphological analysis by means of a scanning electron microscope (SEM; FEI Company, Hillsboro, OR, USA; Quanta 250 FEG). The samples were placed on the adhesive carbon tape. The micrographs were recorded under low vacuum (80 Pa) with an acceleration voltage of 5 kV using secondary electrons collected by a Large Field Detector (LFD).

The morphology of the micelles was observed by means of a transmission electron microscope (TEM) (Tecnai F20 X TWIN; FEI Company) equipped with field emission gun (200 kV acceleration voltage). The micellar solution was placed on a copper grid covered with carbon film, followed by drying at RT. A Gatan Rio 16 CMOS 4k camera (Gatan Inc., Pleasanton, CA, USA) was used for recording the images, and Gatan Microscopy Suite software (Gatan Inc.) was used for processing.

### 2.3. Drug Encapsulation Properties and In Vitro Release Study

The loading content (LC) was calculated from the following equation: LC [%] = (weight of the drug/weight of the drug-loaded NS or M) × 100. The drug-loaded NS or M were dissolved in DMSO and analyzed spectrophotometrically in a Spark 10 M multiplate reader (Tecan, Männedorf, Switzerland): **3a** was analyzed at 298 nm, **3b** at 480 nm, **3c** at 514 nm and **3d** at 303 nm.

#### 2.3.1. Drug Release from NS

Five milligrams of the drug-loaded NS was suspended in 15 mL of phosphate-buffered saline (PBS, pH 7.4) using 15 mL screw-capped tubes and incubated at 37 °C under constant rotation (Loopster digital rotating shaker, IKA, Warsaw, Poland). At the specified time point, the samples were centrifuged (12,000 rpm for 15 min at 20 °C), the supernatants were removed and the precipitate (NS) was saved for analysis of the remaining drug. For this purpose, a modified extraction method was used [11]. Briefly, the NS remaining after each incubation period were dissolved in DMSO and analyzed spectrophotometrically in a Spark 10M multiplate reader (Tecan). The experiment was conducted in triplicate.

#### 2.3.2. Drug Release from Micelles

The dialysis method was used for the analysis of the drug from micelles. The lyophilized micelles (2 mg/mL) were dispersed in PBS (pH 7.4). One milliliter of the micellar solution was placed in a dialysis device (Float-A-Lyzer G2, MWCO of 3.5–5 kDa; Spectra/Por Spectrum Europe B.V., Breda, The Netherlands). Dialysis was conducted against PBS (15 mL). The collected samples were lyophilized, dissolved in DMSO and subjected to quantitative assessment using spectrophotometric measurements in a multiplate reader (Spark^®^, Tecan). The experiment was conducted in triplicate.

### 2.4. Cytotoxicity Study

The cytocompatibility of the blank NS and M was studied according to the ISO 10993-5 standard with the use of the L-929 mouse fibroblast cell line (CCL-1, ™, American Type Culture Collection) [12]. The cytotoxic activity of native drugs, drug-loaded NS and drug-loaded M was studied against the human cervical carcinoma HeLa cell line (CCL-2™, American Type Culture Collection). L-929 and HeLa cells were cultured in Eagle’s Minimum Essential Medium (EMEM) supplemented with 100 U/mL of penicillin, 100 μg/mL of streptomycin and 10% fetal bovine serum. The experimental medium was additionally supplemented with 10 mM HEPES. The cells were maintained at 37 °C in a humidified atmosphere containing 5% CO_2_.

#### 2.4.1. CCK-8 Assay

For analysis of the cytotompatibility of drug-free NS and M and the cytotoxic activity of the free drugs and developed nanoformulations, 100 μL of cell suspension, containing 2 × 10^3^ cells, was transferred to wells of 96-well plates and cultured in standard medium for 24 h. Then, the medium was changed to the medium containing the tested formulations, which were prepared directly before the experiment. The free drug was prepared by dissolution in DMSO (stock solution) and added to the medium to obtain a final concentration range of 3a, 3b, 3c and 3d of 0.4–32 µg/mL. The final concentration of DMSO in the samples was below 0.4%. It has been confirmed that a concentration of DMSO below 1% does not affect the viability of HeLa cells [13,14]. The NS and M were directly suspended in EMEM and diluted in the range of 63.0–500 µg/mL or 2.0–500 µg/mL, respectively. The concentrations of drugs in NS and M are presented in Table 1. The cells were incubated with the tested samples for 72 h. Untreated cells were used as the negative control, and cells treated with 5% DMSO were used as the positive control. The viability of cells was evaluated with the use of Cell Counting Kit–8 (CCK-8). Absorbance was read at 450 nm (reference: 650 nm) in a Spark 10M (Tecan).

#### 2.4.2. Sulforhodamine B Assay

The In Vitro Toxicology Assay Kit, Sulforhodamine B (SRB)-based (Sigma Aldrich, St. Louis, MO, USA), was used to study the viability by measuring total biomass through staining cellular proteins with SRB. The HeLa cells were seeded in 96-well plates at a density of 2 × 10^3^ per well in 100 μL of medium and cultured for 24 h. Then, the medium was removed and replaced with a fresh one (200 μL), containing blank micelles at concentrations of 64, 125, 250 and 500 μg/mL or 3d-loaded micelles (M/3d) at a concentration range of 2–500 μg/mL. The cells were exposed to the tested samples for 72 h. Then, the medium was removed, and the cells were fixed with 10% trichloroacetic acid and stained with SRB. The absorbance was read at 570 and 690 nm (reference wavelength) using a microplate reader (Spark 10 M; Tecan).

#### 2.4.3. LDH Assay

The cytotoxicity of M/3d was also analyzed by measuring the activity of lactate dehydragenase (LDH) (Cytotoxicity LDH Assay Kit). The cell culture was prepared in the same way as for the CCK-8 assay. Apart from cells cultured with M/3d at a concentration of 2–500 μg/mL for 24 h, two types of control groups were prepared. One set of cell culture wells was lysed by the addition of 10 μL of Lysis Solution to determine the maximum LDH release. The second set of cell culture wells was used to determine the spontaneous LDH release by the addition of 10 μL of medium. The percentage of cytotoxicity was determined by the following equation: Cytotoxicity (%) = [(X − Z)/(Y − Z)] × 100%, where X: Absorbance of Samples − Background Blank; Y: Absorbance of High Control − High Blank Control; Z: Absorbance of Low Control − Background Blank

#### 2.4.4. BrdU Assay

Analysis of the rate of DNA synthesis was conducted with the use of the bromodeoxyuridine (BrdU) incorporation assay (Cell Proliferation ELISA, BrdU colorimetric kit; Roche, Basel, Switzerland). HeLa cells were cultured in 96-well plates (4 × 10^3^ per well) under standard conditions for 24 h. Subsequently, the medium was removed and replaced with medium containing blank micelles (at concentrations of 64, 125, 250 and 500 μg/mL) or 3d-loaded micelles (M/3d) at a concentration range of 2–500 μg/mL. The cells were incubated with the tested samples for 72 h, then the culture medium was removed, and the cells were treated with FixDenat to denature DNA. Absorbance was measured at 370 and 492 nm (reference wavelength) with the use of a microplate reader (Spark 10 M; Tecan) to evaluate the amount of BrdU retained in cells.

### 2.5. Hemolysis Assay

A hemolysis assay was conducted for drug-free NS (NS), drug-free micelles (M), drug-loaded NS (NS/3a, NS/3b, NS/3c and NS/3d) and drug-loaded micelles (M/3a, M/3b, M/3c and M/3d) according to the procedure described by Sæbø et al. [15]. The study was conducted after receiving approval from the Bioethical Commission of the Medical University of Silesia (No. BNW/NWN/0052/KB1/25/24). A 10% Triton X-100 solution was used as a positive control (C+) and PBS (pH ~7) as a negative control (C−) in identical volumes as the test compounds. The blood from healthy volunteers was collected in lithium heparin tubes and centrifuged at 1700× *g* for 5 min. The supernatant was removed by aspiration, and erythrocytes were washed with the addition of 2 mL of PBS (pH ~7) and centrifuged. The washing step was repeated three times until the supernatant was clear. Then, the supernatant was removed, and the erythrocyte pellet was diluted 1:100 in PBS (pH ~7) to obtain a 1% erythrocyte suspension. A 500 μL erythrocyte suspension was mixed with 500 μL of the tested compound and incubated at 37 °C for 60 min. The samples were centrifuged, and 250 μL of the supernatant was transferred to a transparent, flat-bottom 96-well plate to measure absorbance at 405 nm in a Spark 10 M microplate reader (Tecan).

### 2.6. Statistical Analysis

The results were analyzed using a one-way ANOVA followed by a Tukey post hoc test. A *p*-value of <0.05 was considered statistically significant.

## 3. Results

### 3.1. Characteristics of the NPs

**Figure 1:**
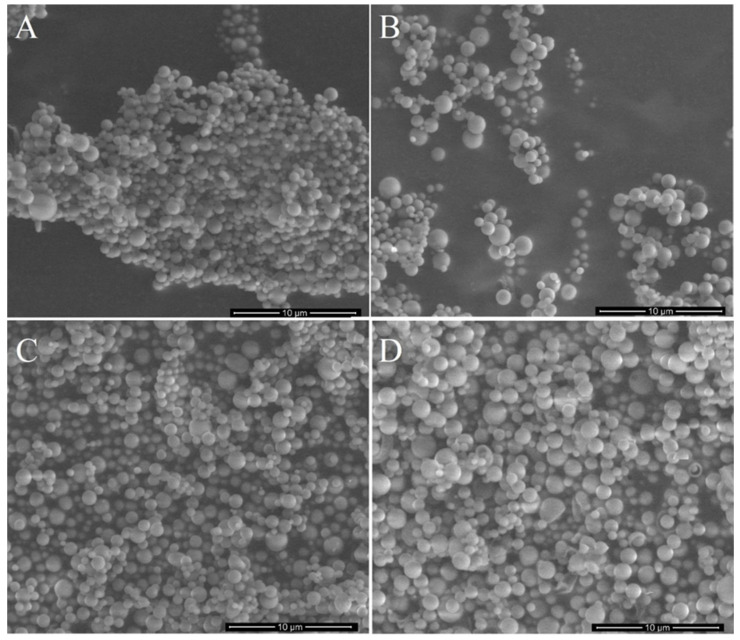
SEM images of nanospheres loaded with **3a** (**A**), **3b** (**B**), **3c** (**C**) and **3d** (**D**).

**Figure 2:**
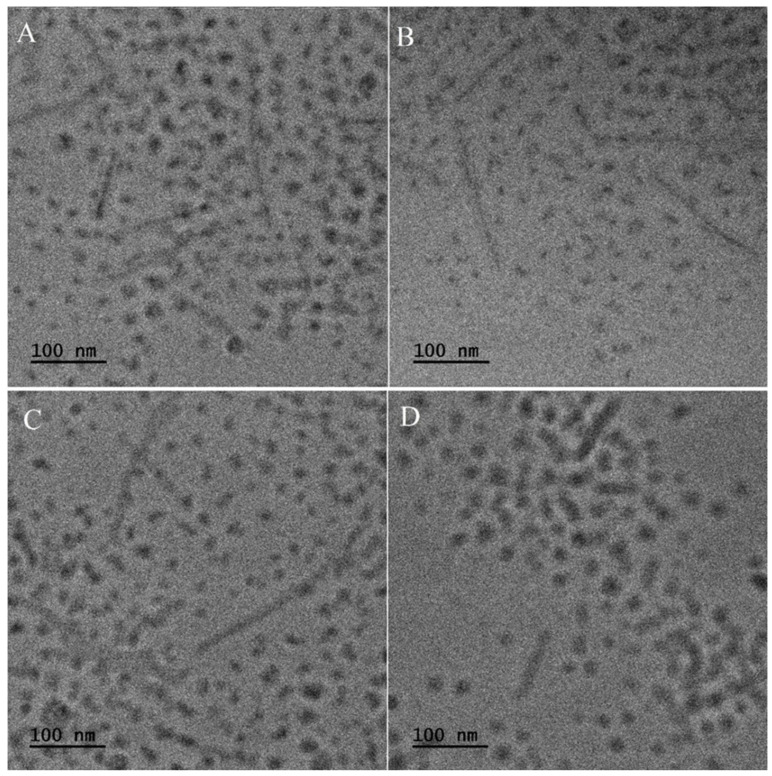
TEM images of micelles loaded with **3a** (**A**), **3b** (**B**), **3c** (**C**) and **3d** (**D**).

Two types of NPs with phenothiazine derivatives have been developed: nanospheres and micelles. The morphology of NS loaded with **3a**, **3b**, **3c** and **3d**, observed in SEM, is presented in Figure 1. All types of NS are characterized by very regular spherical shapes with a smooth surface and an average diameter of ≈870 nm. Thus, it may be concluded that the size and morphology of NS do not depend on the type of encapsulated active compound. Similarly, no differences in size and morphology were observed for phenothiazine derivative-loaded micelles (Figure 2). In all cases, two types of micelles were formed, with a spherical and elongated shape. The micelles possess a diameter of ≈25 nm, and the length of the elongated nanocarriers is ≈150 nm.

### 3.2. Drug-Loading and Release Properties

The loading content of 3a, 3b, 3c and 3d in nanospheres and micelles is presented in Table 2. It can be observed that the drug-loading properties differ significantly depending on the type of NP. Generally, the LC of all phenothiazine derivatives in NS is significantly lower than the LC of micelles. The highest loading capacity was observed for NS with **3d** (3.07%) and the lowest for **3a** (0.75%). In the case of micelles, the LC is similar for **3a**, **3c** and **3d** (≈21%) and slightly lower for **3b** (≈18%).

**Figure 3:**
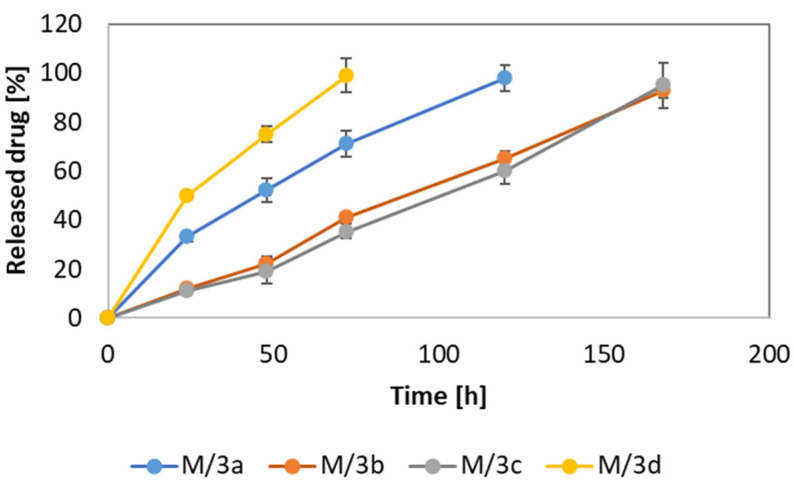
Comparison of drug release from various micellar formulations.

Drug release was studied in an in vitro environment. The drug release from NS was very insignificant and below detection even after 30 days of incubation. The release process from micellar formulations proceeded much faster, as presented in Figure 3, because after 24 h, 50% of the drug was released from M/3d and 33% from M/3a. The release of drug was completed after 72 h from M/3d and after 120 h from M/3a. The slowest release was observed for M/3b and M/3c, because it was extended to 168 h. No significant differences in drug release were observed between M/3b and M/3c; however, differences were observed in other samples. Additionally, significant differences were observed between M/3a and M/3d. The differences in the rate of release of particular phenothiazine derivatives may suggest the influence of the type of substituent in the 9, 10 and 11 positions of the quino[3,4-*b*][1,4]benzothiazine scaffold. The derivative with OH groups in the quinobenzothiazinium system (**3d**) showed the most rapid drug release.

### 3.3. Hemolytic Effect

**Figure 4:**
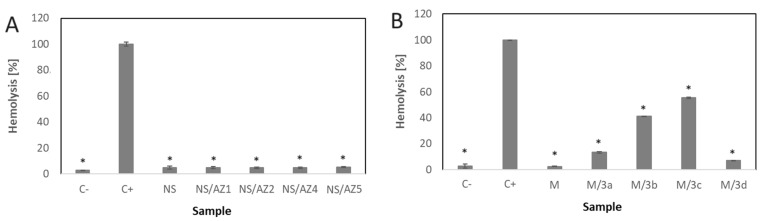
Hemolytic effect of nanospheres (**A**) and micelles (**B**) (* vs. C+, ±SD, *n* = 3).

A hemolytic assay was conducted to evaluate the hemocompatibility of the developed formulations. Hemolysis is defined as rupturing the membrane of erythrocytes, which causes the release of hemoglobin and other internal components into the surrounding fluid [16]. The hemolytic effect assay is presented in Figure 4. Out of all types of NS, both the drug-loaded (NS/3a, NS/3b, NS/3c and NS/3d) and drug-free (NS) formulations did not show any hemolytic effect (Figure 4A). In contrast, differences were observed in the effect of micelles on the erythrocytes (Figure 4B). Drug-free micelles (M) showed effects similar to those of the negative control. Insignificant hemolytic effects were exhibited by micelles loaded with **3a** (M/3a) and **3d** (M/3d). Significantly increased hemolysis was observed for M/3b and M/3c.

### 3.4. Cytotoxic Activity

**Figure 5:**
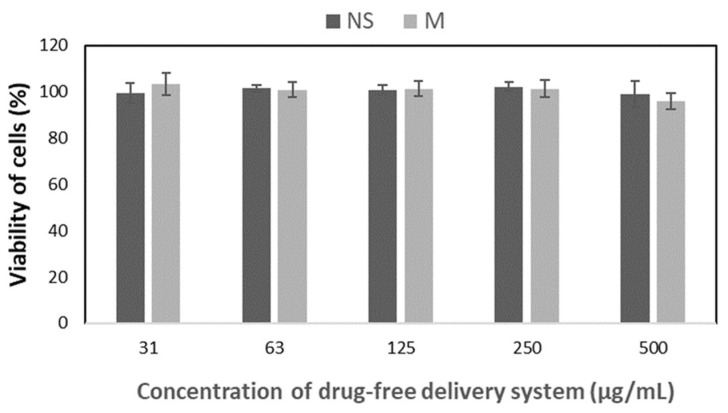
The effect of the drug-free nanospheres and micelles on the viability of L-929 cells (the results are shown as mean ± SD; *p* < 0.05 compared with control).

The in vitro cytotocompatibility of the drug-free nanospheres and micelles was analyzed according to the ISO 10993-5 standard with the use of L-929 fibroblasts and a CCK-8 assay. The sensitive colorimetric CCK-8 assay is used for the evaluation of cell viability. It uses 2-(2-methoxy-4-nitrophenyl)-3-(4-nitrophenyl)-5-(2,4-disulfophenyl)-2H tetrazolium, monosodium salt (WST-8), which is reduced to an orange formazan product by cellular dehydrogenases, and the produced formazan is directly proportional to the number of live cells. As shown in Figure 5, proliferation in all tested drug-free formulations did not differ from that in the standard cell culture, which confirms the cytocompatibility of all studied drug-free nanoparticles.

**Figure 6:**
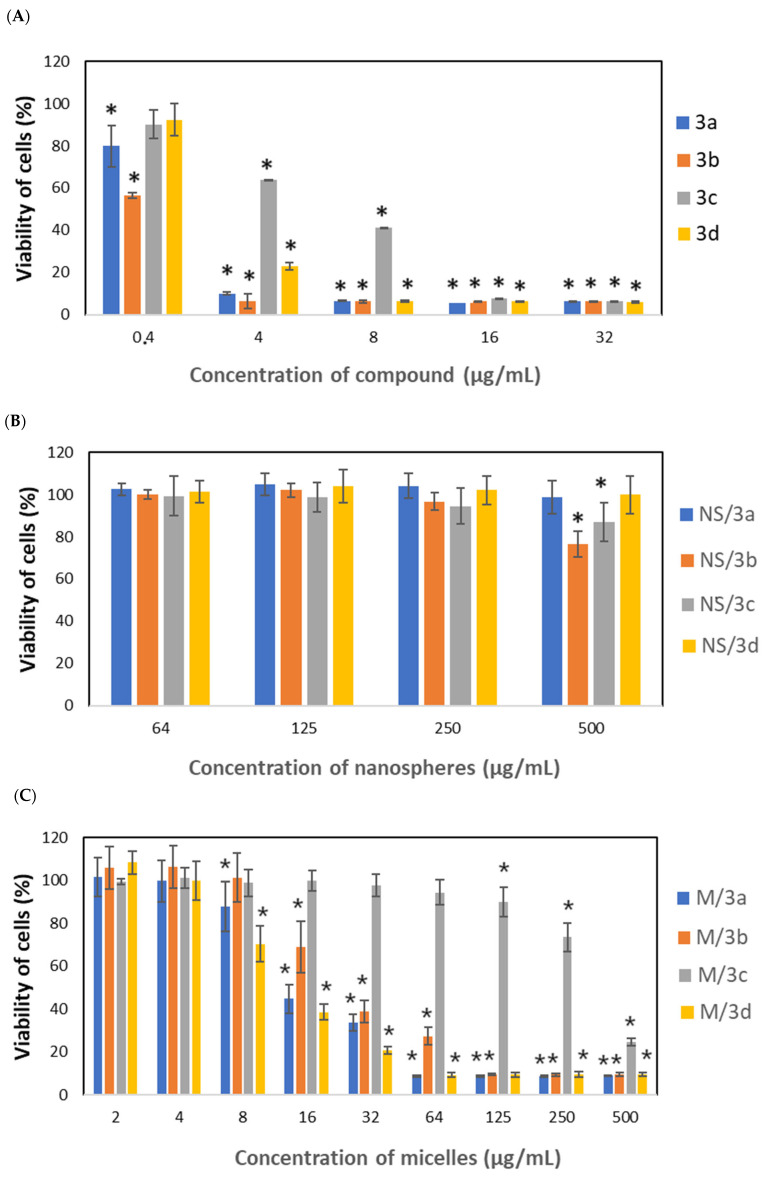
The effect of native compounds (**A**), compounds loaded into nanospheres (**B**) and compounds loaded into micelles (**C**) on the viability of HeLa cells, determined by means of a CCK-8 assay (the results are shown as mean ± SD; * *p* < 0.05 compared with control).

In the next step, a cytotoxicity study was conducted to evaluate the effect of the native drug, the drug encapsulated in NS and the drug encapsulated in M against cervical cancer cells (HeLa) (Figure 6). In line with expectations, the strongest effect was observed for the free-drug formulation, which caused a decrease in cell viability even at the lowest studied concentration (0.4 µg/mL), as observed in the case of 3a and 3b (Figure 6A). At concentrations equal to and greater than 4 µg/mL, all compounds showed a cytotoxic effect; however, the effect was less significant for 3c at 8 and 16 µg/mL.

The drugs encapsulated in NS showed a significantly slighter cytotoxic effect on HeLa cells (Figure 6B). A decrease in cell viability was observed only for NS/3b and NS/3c at 500 µg/mL.

A significantly stronger cytotoxic effect was observed for drug-loaded micelles (Figure 6C). Inhibition of cell growth in the presence of M/3a and M/3d was observed at a concentration range of 8–500 µg/mL and M/3b from 16 to 500 µg/mL. The lowest inhibitory effect was exhibited by M/3c (125–500 µg/mL).

**Figure 7:**
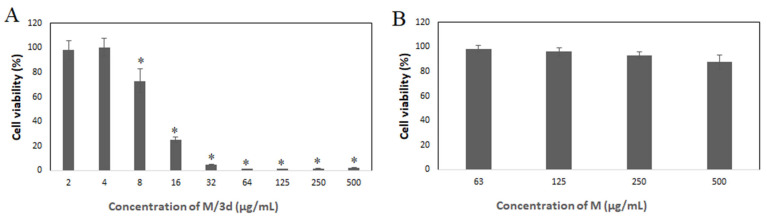
The effect of the 3d compound loaded into micelles (**A**) and drug-free micelles (**B**) on the viability of HeLa cells, analyzed by means of an SRB assay (the results are shown as mean ± SD; * *p* < 0.05 compared with control).

An additional cytotoxicity study was conducted for selected types of nanoparticles: M/3d. The decrease in viability of HeLa cells cultured in the presence of M/3d at concentrations equal to or greater than 8 µg/mL was confirmed by an SRB assay (Figure 7A). The drug-free micelles did not affect the viability of cells, as shown in Figure 7B.

**Figure 8:**
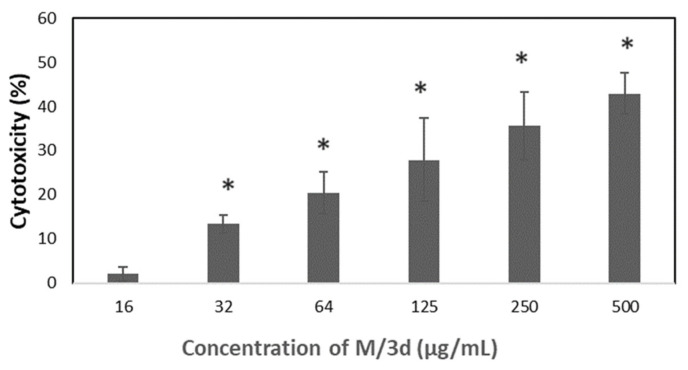
The effect of M/3d on LDH release from HeLa cells (the results are shown as mean ± SD; * *p* < 0.05 compared with control).

M/3d at a concentration equal to or greater than 32 µg/mL caused a significant increase in LDH release, as presented in Figure 8.

**Figure 9:**
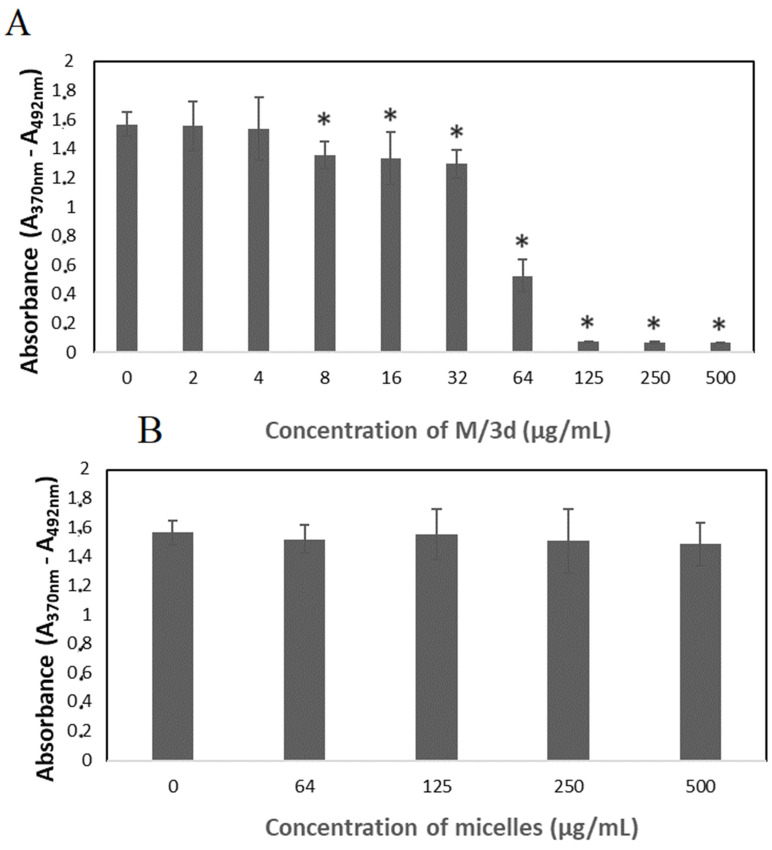
The effect of the 3d compound loaded into micelles (**A**) and drug-free micelles (**B**) on the proliferation of HeLa cells, determined by means of a BrdU assay (the results are shown as mean ± SD; * *p* < 0.05 compared with control).

The bromodeoxyuridine (BrdU) incorporation assay was conducted to analyze the synthesis of DNA in cells treated with M/3d. The results show that M/3d, at concentrations of 8 µg/mL and higher, caused a significant reduction in cell division (Figure 9A). Cell division was not affected in the presence of blank micelles (Figure 9B), confirming their biocompatibility and the fact that the cytotoxic effect was caused by the 3d compound released from the micelles.

## 4. Discussion

Cancer is the second leading cause of death, and this burden continues to increase. Conventional cancer treatments often suffer from limitations such as systemic toxicity, poor pharmacokinetics and drug resistance. Therefore, there is an urgent need for novel drugs with increased efficacy for the treatment of different cancers [17,18]. In our previous study, new 5-methyl-12*H*-quino[3,4-*b*][1,4]benzothiazinium chlorides were synthesized and characterized (Scheme 1). The anticancer activity of the compounds was studied in vitro against human colon carcinoma and Lewis lung carcinoma cell lines [2]. The greatest cytotoxic activity was observed for compounds with substituents at the 9- and 10-positions of the quinobenzothiazine ring and for compounds with no substituents. Despite their therapeutic potential, the new compounds display some drawbacks that could reduce their effectiveness, e.g., limited solubility in water. Therefore, along with the development of novel compounds with anticancer potential, it is also necessary to develop an appropriate DDS. To overcome these limitations, various DDSs have been developed, and one with nanotechnology being a leading approach. Nanoparticles are carriers of submicron size that can be created to contain and deliver therapeutic substances to specific locations in the body, including tumors. Compared to traditional chemotherapy, nano-delivery has many benefits, such as excellent drug solubility, prolonged drug release and enhanced cellular uptake, leading to increased efficacy and reduced toxicity [18].

Two types of nanoparticles were selected to evaluate their potential in the delivery of phenothiazine derivatives to cancer cells: nanospheres and micelles. NS are defined as homogeneous matrix systems wherein a dispersed or dissolved active compound is entrapped within the polymeric matrix structure through the solid sphere [19]. Loading of drug(s) into the NS enables local drug delivery because, during a specific time period, these nanostructures undergo slow hydrolytic degradation, disintegration of the matrix and sustained release of the drug. NS have a small particle size, and thus, they are suitable to be administered orally, locally, and systemically. Most NS are prepared using polymers that are biodegradable and biocompatible. Currently, NP obtained from poly(lactide-co-glycolide) (PLGA), a U.S. FDA-approved biocompatible polymer, has been widely explored [20]. Aliphatic polyesters characterize biocompatibility and tailorable degradation rate (e.g., by molar mass, composition and copolymer microstructure). The main advantage of aliphatic polyesters such as PLGA is their hydrolytic degradation into products that are naturally present in the human body (e.g., lactic acid and glycolic acid) [21].

Polymeric micelles are another example of nanocarriers extensively studied for anticancer drug delivery, and some have already been applied at different stages of clinical trials [22]. Polymeric micelles are particles of 5–200 nm formed by the self-assembling of amphiphilic polymers. They consist of a hydrophobic core (which can serve as a solubilization depot for agents with poor aqueous solubility) and hydrophilic shell, providing increased stability in the blood and longer blood circulation time [13,22,23]. Compared to surfactant micelles, polymeric micelles provide higher stability, greater possibility for controlling structure and functionality by modification of polymer composition, structure and molar mass [24]. In particular, micelles obtained from poly(lactide)-polyethylene glycol (PLA–PEG) have been considered for drug delivery because the PEG prevents it from adsorption by proteins and phagocytes, which increases blood circulation time, and the PLA core is responsible for encapsulating a hydrophobic drug [22,25,26]. Micelles’ advantages involve easy processability, effective drug solubilization, improved biocompatibility, pharmacokinetics, and biodistribution [27].

The obtained nanoparticles have been characterized for their morphology and drug encapsulation properties. It has been determined that the type of active agent (phenothiazine derivative) did not affect the nanoparticles’ morphology, because all types of NS possess the same regular spherical shape with a smooth surface and an average diameter of ≈870 nm (Figure 1). Similarly, no differences in size and morphology have been observed for phenothiazine derivatives-loaded micelles (Figure 2). Significant differences have been observed in drug loading properties between NS and M because the content of all kinds of active compounds was much higher in the case of micelles (Table 2). Apparently, the matrix-type of DDS, typical for NS, was not efficient for encapsulation of phenothiazine derivatives. Additionally, despite both kinds of drug-free nanoparticles showing cytocompatibility evaluated according to the ISO 10993-5 standard (Figure 5), the drug-loaded NS did not show any cytotoxic effect against HeLa cancer cells at a concentration below 500 µg/mL. Moreover, even at a concentration of 500 µg/mL, only NS/3b and NS/3c showed some cytotoxicity against HeLa cells. The reason for such insignificant cytotoxic effect of drug-loaded NS may be their low drug content (Table 2) and negligible drug release. In contrast, the drug-loaded micelles showed cytotoxic effect against cancer cells at much lower concentrations (Figure 6C), because inhibition of cell growth in the presence of M/3a and M/3d was observed at a concentration of 8 µg/mL, M/3b from 16 µg/mL and M/3c from 125 µg/mL. This effect may have been facilitated by the high drug content of the drug in micelles (≈20%) (Table 2) and their faster release, which reached 50% in the case of M/3d.

In the next step, the hemolytic effect was evaluated for NS and M, which also influences their pharmaceutical potential. Determination of hemolytic properties in vitro is a common and important method for the preliminary evaluation of the cytotoxicity of various materials, chemicals, drugs or any blood-contacting medical devices [15]. As presented in Figure 4, all types of NS, including both the drug-loaded and drug-free formulations, did not show any hemolytic effect (Figure 4A). On contrast, differences were observed in the effect of micelles on the erythrocytes (Figure 4B). Drug-free micelles (M) showed an effect similar to the negative control (C−). The M/3a and M/3d caused a very insignificant hemolytic effect. Significantly increased hemolysis was observed for M/3b and M/3c. Apparently, the hemolytic effect was caused by the released active agent (3a–3d). In fact, the hemolysis of human erythrocytes by certain phenothiazine drugs and phenothiazine derivatives has been reported [28,29,30].

Based on the preliminary study, the 3d-loaded micelles (M/3d) were selected for more detailed analysis of cytotoxicity against cancer cells. In fact, M/3d showed the highest drug-loading content, the most rapid release and the lowest hemolytic activity, making them the most promising formulation. The SRB assay confirmed the decrease in cell viability above 8 µg/mL of M/3d (Figure 7A). The cytotoxicity of M/3d was also analyzed by measuring the activity of lactate dehydrogenase (LDH). LDH is a stable enzyme, present in all cell types, that is rapidly released into the cell medium upon damage to the plasma membrane, making it a useful marker in cytotoxicity studies. M/3d concentrations above 32 µg/mL caused significantly increased LDH release (Figure 8). The effect of M/3d on DNA synthesis was analyzed by means of a BrdU-incorporation assay. It was observed that M/3d at a concentration of 8 µg/mL and higher caused a significant reduction in cell division (Figure 9A). The effect of drug-free micelles did not affect HeLa cell viability and proliferation (Figure 7B and Figure 9B), confirming their biocompatibility and the fact that the cytotoxic effect was caused by the 3d compound released from the micelles.

## 5. Conclusions

PLA-based nanospheres and micelles were evaluated as delivery systems for four types of 5-methyl-12*H*-quino[3,4-*b*][1,4]benzothiazinium chlorides with anticancer potential. The analyzed compounds (**3a**–**d**) differed in the type of substituents (CH_3_, NH_2_, OH) (Scheme 1). The micelles appeared much more efficient at loading active compounds and their release, which caused significantly higher cytotoxic effect against cancer cells than that observed for nanospheres. Thus, despite the cytocompatibility of both types of nanoparticles, only micelles confirmed their feasibility as carriers of phenothiazine derivatives. The micelles containing **3d**—the compound with an OH group as a substituent in the 10-position of the quinobenzothiazine ring—showed the highest drug-loading content, the most efficient drug release, the lowest hemolytic activity and the most significant cytotoxic effect against cancer cells. Therefore, M/3d has been selected as the most promising formulation for anticancer application. The conducted study enabled the development of a delivery system for the new anticancer compound and showed that the choice of drug carrier has a crucial effect on its cytotoxic potential against cancer cells.

## Figures and Tables

**Table 1:** Concentration of drugs in the medium containing drug-loaded nanospheres and micelles.

Concentration of Drug-Loaded NS or M (µg/mL)	NS/3a	NS/3b	NS/3c	NS/3d	M/3a	M/3b	M/3c	M/3d
2	N/A	N/A	N/A	N/A	0.42	0.36	0.41	0.42
4	N/A	N/A	N/A	N/A	0.84	0.72	0.82	0.83
8	N/A	N/A	N/A	N/A	1.69	1.44	1.65	1.67
16	N/A	N/A	N/A	N/A	3.39	2.89	3.30	3.34
31	N/A	N/A	N/A	N/A	6.79	5.78	6.60	6.68
63	0.16	1.71	1.13	1.92	13.58	11.56	13.21	13.37
125	0.31	3.42	2.27	3.74	27.17	23.13	26.43	26.75
250	0.62	6.85	4.55	7.67	54.35	46.27	52.87	53.50
500	1.25	13.70	9.10	15.35	108.70	92.55	105.75	107.00

**Table 2:** Drug-loading capacity of the nanospheres and micelles.

Name of Drug Delivery System	LC (%)
NS/3a	0.75 ± 0.07
NS/3b	2.74 ± 0.13
NS/3c	1.82 ± 0.10
NS/3d	3.07 ± 0.17
M/3a	21.74 ± 1.34
M/3b	18.51 ± 0.49
M/3c	21.15 ± 0.45
M/3d	21.40 ± 1.11

## Data Availability

The original contributions presented in this study are included in the article. Further inquiries can be directed to the corresponding authors.
